# Incessant Refractory Polymorphic Ventricular Tachycardia After Coronary Artery Bypass Graft

**DOI:** 10.7759/cureus.12752

**Published:** 2021-01-17

**Authors:** Michele M Iguina, Shaun Smithson, Mauricio Danckers

**Affiliations:** 1 Internal Medicine, Aventura Hospital and Medical Center, Aventura, USA; 2 Cardiology, Aventura Hospital and Medical Center, Aventura, USA; 3 Critical Care Medicine, Aventura Hospital and Medical Center, Aventura, USA

**Keywords:** coronary artery bypass grafting(cabg), ventricular tachycardia, coronary artery angiography, polymorphic ventricular tachycardia

## Abstract

Polymorphic ventricular tachycardia (PVT) post coronary artery bypass (CABG) surgery is associated with acute myocardial ischemia, hemodynamic instability, and metabolic derangements. When acute ischemia is suspected, a comprehensive investigation for reversible causes is justified to improve patient outcomes. We present a curious case of incessant, refractory PVT in a patient with an unknown etiology requiring percutaneous coronary intervention (PCI) post CABG. The patient was a 73-year-old female with multiple comorbidities who presented to the hospital with anginal chest pain for one day. Initial electrocardiogram (EKG) showed sinus tachycardia with ST-segment depressions in the inferior-lateral leads. Initial cardiac troponin I was elevated at 28.280 ng/mL. Dual antiplatelet therapy and heparin were started. Urgent coronary angiography revealed significant triple-vessel disease, and she subsequently underwent three-vessel CABG. Her postoperative course was complicated by PVT refractory to all antiarrhythmic therapy and ventricular fibrillatory (VF) arrest with the recovery of spontaneous circulation after defibrillation and amiodarone bolus. Despite normal electrolytes and discontinuation of all QT-prolonging agents, PVT persisted. Urgent coronary angiography revealed a patent venous graft to a previously underappreciated severely stenotic distal segment of the left anterior descending artery (LAD). She underwent PCI of the culprit lesion with the termination of PVT. Although acute graft failure is regularly the culprit for acute myocardial infarction perioperatively, emergent coronary angiography post coronary bypass surgery revealed patent grafts and a previously underestimated severe coronary lesion contributing to ongoing ischemia. Post CABG percutaneous coronary intervention (PCI) yielded a complete resolution of her arrhythmia.

## Introduction

Perioperative polymorphic ventricular tachycardia (PVT) after coronary artery bypass graft (CABG) surgery can be life-threatening and warrants thorough investigation for reversible causes [1]. PVT is a rapid, unstable ventricular arrhythmia defined by variable QRS complex morphology on electrocardiogram (EKG). Etiologies include congenital defects in myocardial repolarization, electrolyte abnormalities, drug toxicities, and ischemic myocardial injury. Once identified, it warrants immediate medical intervention for prompt reversal and prevention. Acute myocardial ischemia is the most commonly cited etiology for PVT post CABG. Although graft failure is the most common cause of perioperative myocardial infarction, we present a case of refractory PVT in a post CABG patient with an unsuspected etiology of coronary ischemia requiring post-CABG percutaneous coronary intervention (PCI).

## Case presentation

**Figure 1:**
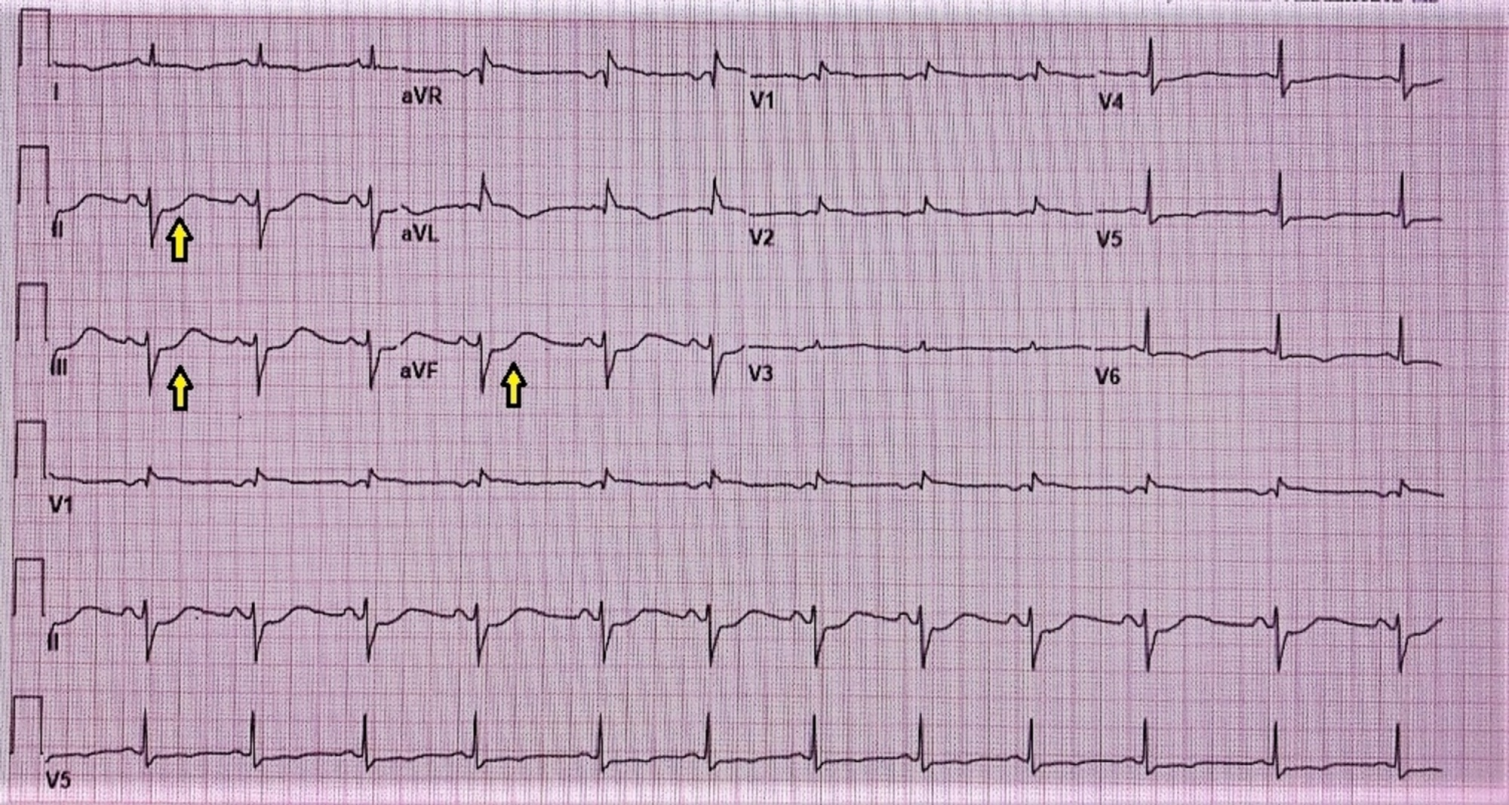
EKG: ST-segment depressions in inferior leads EKG: electrocardiogram

**Figure 2:**
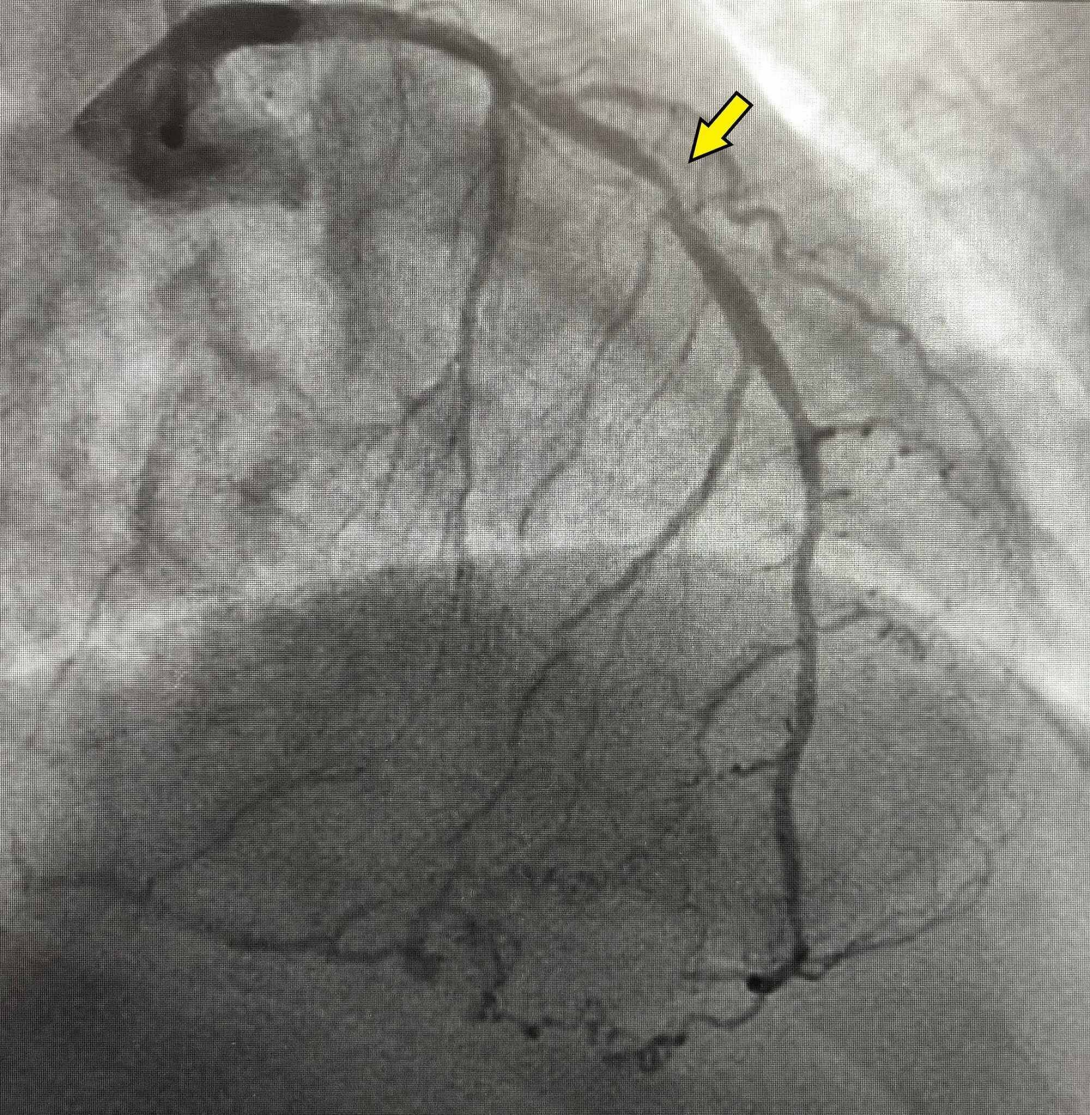
Coronary angiography pre coronary artery bypass graft (CABG) showing severe stenosis of proximal left anterior descending (LAD) artery

**Figure 3:**
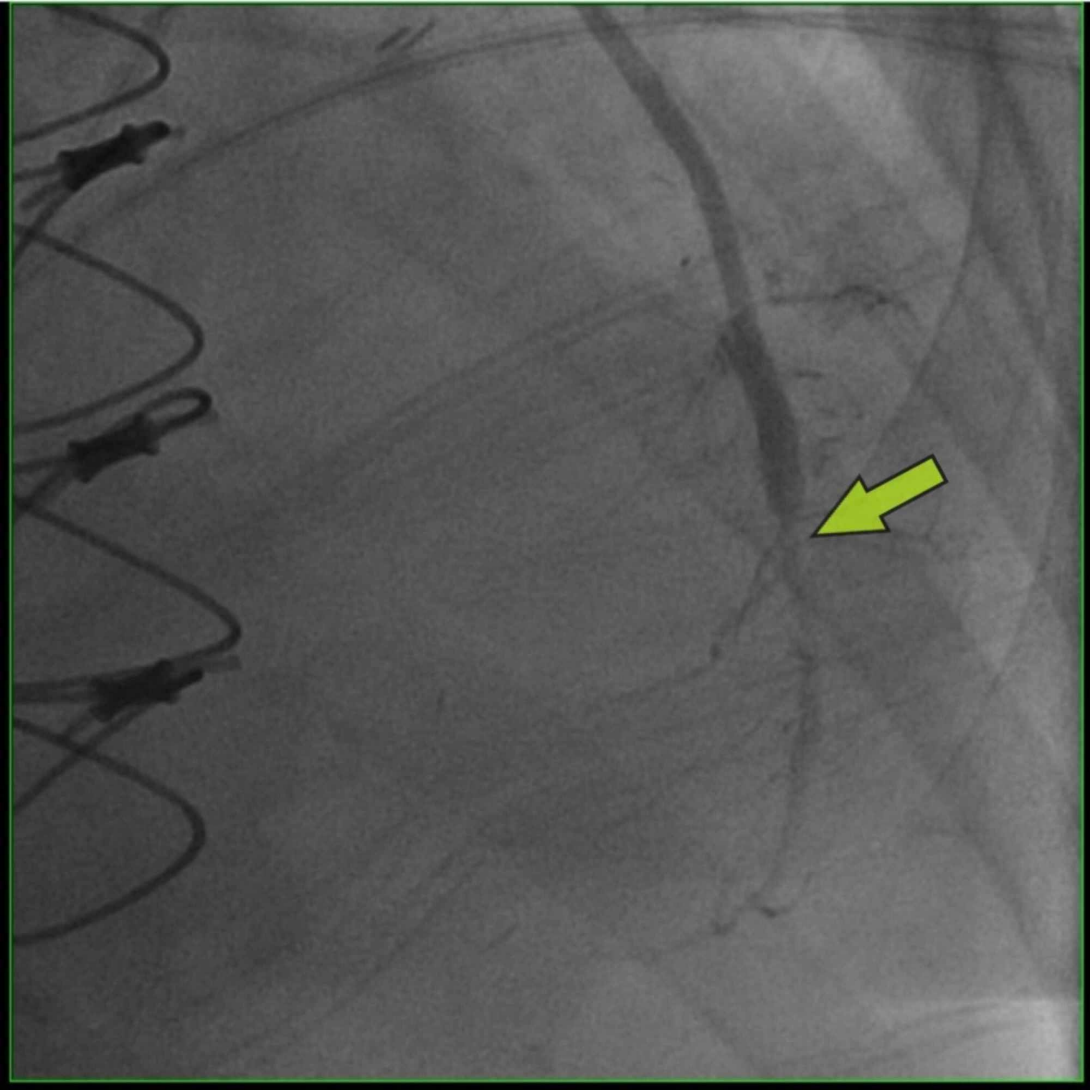
Coronary angiography post coronary artery bypass graft (CABG) showing patent left internal mammary artery (LIMA) graft to left anterior descending (LAD) artery with severe distal stenosis after the graft insertion site

A 73-year-old female presented to the hospital with chest pain for one day. Initial EKG showed ST-segment depressions in the inferior-lateral leads (Figure 1). Cardiac troponin I was elevated at 28.280 ng/ml. Urgent coronary angiography revealed total occlusion of the proximal left circumflex (LCx), subtotal occlusion of the proximal right coronary artery (RCA), and severe stenosis of the proximal left anterior descending (LAD) artery (Figure 2). She underwent a three-vessel bypass CABG. Her postoperative course was complicated by prolonged vasoplegia and hemodynamic instability requiring high-dose vasopressors. Three days post-CABG, PVT ensued refractory to ventricular pacing, electrolyte replacement, and intravenous amiodarone, magnesium, and lidocaine infusion. Telemetry revealed PVT associated with the prolongation of the QTc interval at 560 ms and with morphology compatible with torsade de pointes (TdP). Suspected to be acquired TdP, intravenous amiodarone and lidocaine were discontinued, but PVT persisted. Electrolytes including potassium and magnesium were within normal limits at 4.2 mmol/L and 2.0 mg/dL respectively. Five days post CABG, she developed ventricular fibrillation (VF) arrest, and spontaneous circulation was recovered after defibrillation and amiodarone bolus. Urgent cardiac angiography revealed a patent graft to a severely stenotic distal segment of the LAD (Figure 3). She underwent PCI of the culprit lesion with the termination of PVT. She was eventually discharged home on post-op day 25.

## Discussion

Acute myocardial ischemia is the most commonly cited etiology for PVT post-CABG, and treatment should be guided at reperfusion. Other known etiologies include electrolyte abnormalities, increased sympathetic activity, hemodynamic instability, and acquired or congenital QT prolongation. According to several retrospective observational studies, perioperative PVT typically occurs within the first week of surgery [2-4]. In our case, PVT manifested 72 hours post-CABG, a time when the patient was still requiring multiple vasoactive medications secondary to prolonged vasoplegia. Ventricular pacing was attempted in an effort to prevent pause dependent arrhythmia - a class I recommendation per the 2012 update of the American College of Cardiology/American Heart Association/Heart Rhythm Society (ACC/AHA/HRS) guidelines for device-based therapy of cardiac rhythm abnormalities [5]. Despite addressing metabolic derangements and the cessation of antiarrhythmics, PVT persisted. Recurrent ventricular tachycardia that persists for more than 12 hours refractory to repeated attempts to terminate the arrhythmia is termed “incessant” [6]. PVT frequently degenerates into ventricular fibrillation (VF) and requires emergent defibrillation.

The suspicion for ongoing myocardial ischemia was high in our patient in lieu of incessant refractory PVT and VF arrest. Two small series reported approximately 3% to 6% of post-CABG patients to develop clinically apparent postoperative ischemia as manifested by ischemic symptoms, ischemic ECG abnormalities, hemodynamic instability, and/or ventricular arrhythmias [7-8]. One of the most common etiologies of perioperative myocardial infarction includes graft failure, reported in up to 12% of grafts [9-10]. However, in our case, urgent coronary angiography revealed a patent graft with severe LAD stenosis distal to the graft insertion site, ultimately requiring emergent PCI with a resolution of the PVT. Prior studies have reported a tendency of coronary angiography to underestimate the severity of lesions affecting the left main coronary artery and its branches, especially the LAD [11-12]. Borren et al. reported 30% of all LAD lesions were angiographically underestimated compared with 11% of all non-LAD lesions [13]. Therefore, PVT post CABG should trigger an ischemic workup and necessitates reperfusion-guided therapy for resolution.

The days after cardiac surgery can be thought of as a “stress test,” full of potentially reversible causes of ventricular arrhythmias [14]. Post-CABG myocardial injury and its secondary complications remain an utmost concern [15]. Early graft failure would be the most expected etiology of our patient’s recurrent ischemia. However, despite patent grafts, incomplete revascularization of her native distal LAD lesion prompted post-CABG angiography and PCI with the resolution of her arrhythmia. PCI post-CABG is a paradigm shift in revascularization strategies and is more common than previously predicted, per Alqahtani et al. [16]. Investigators found that patients who undergo PCI post-CABG have higher rates of stroke (2.1% vs 1.6%), acute kidney injury (16.0% vs 12.3%), and unadjusted in-hospital mortality (5.1% vs 2.7%) vs. the non-PCI group [16]. Despite these adverse events, PVT post-CABG is an ischemic arrhythmia that warrants diagnostic angiography and intravascular intervention guided at treating any reversible etiologies.

## Conclusions

Despite scientific advances in coronary angiography, stent technology, and cardiothoracic surgery techniques, perioperative myocardial injury post CABG remains a significant concern. PVT post CABG carries high mortality if not treated with urgency. Although emergent PCI post CABG resulted in a positive outcome in our patient, current evidence suggests this clinical scenario carries alarming outcomes for both patients and the healthcare system. Further research in identifying the predictors of coronary compromise post CABG is warranted to improve outcomes in this high-risk patient population.
